# Gross anatomy, computed tomographic contrast tenography, and needle endoscopy of the equine medial digital flexor tendon sheath

**DOI:** 10.1111/vsu.14263

**Published:** 2025-04-14

**Authors:** Maria P. Kerbert, Uta Delling, Nicole Verhaar

**Affiliations:** ^1^ Clinic for Horses University of Veterinary Medicine Hannover Hannover Germany

## Abstract

**Objective:**

To investigate the computed tomography (CT) and gross anatomy of the equine medial digital flexor tendon sheath (MDFTS), and the endoscopic anatomy and approach to the MDFTS.

**Study design:**

Ex vivo experimental study and one clinical case.

**Sample population:**

Twelve clinically normal cadaveric hindlimbs.

**Methods:**

Dissection, native and contrast CT scans were conducted to evaluate the anatomy of the MDFTS. Based on these findings, the portal locations for the endoscopic approaches were determined. Six hindlimbs were used in the pilot phase and another six limbs were selected for the main study. Endoscopic images were reviewed by three observers for the quality of visualization of intrasynovial structures during endoscopy.

**Results:**

Intrasynovial structures that could consistently be identified during dissection and CT examination included the mesotenon in the proximal recess and two synovial plicae surrounding the medial digital flexor tendon. Communication between MDFTS and tarsal sheath varied among individual horses. Two portals were necessary to visualize the complete length of the MDFTS. Endoscopic entry was possible with both a needle scope and a conventional arthroscope; however, endoscopic examination was only feasible with the needle scope. The aforementioned intrasynovial structures could also be visualized endoscopically. One clinical case is presented with septic tenosynovitis due to a laceration with penetration of the MDFTS.

**Conclusion:**

Endoscopic examination of the MDFTS is possible with the use of a needle scope.

**Clinical significance:**

Endoscopic lavage of the MDFTS might be indicated in cases of septic tenosynovitis of the MDFTS and/or tarsal sheath.

## 1 INTRODUCTION

The deep digital flexor tendon in the hindlimb consists of three structures: the lateral digital flexor tendon (*M. flexor digitalis lateralis* ^1^, LDFT), the caudal tibial tendon (*M. tibialis caudalis* ^1^, CTT) and the medial digital flexor tendon (*M. flexor digitalis medialis* ^1^, MDFT).^2^ The CTT fuses with the LDFT just proximal to the tarsal sheath.^3^ The MDFT runs just plantar to the long superficial medial collateral ligament of the talocrural joint (TCJ)^4^ and passes through the groove in the proximal tubercle of the talus (*Tuberculum tali proximalis* ^1^, PTT).^3^ The MDFT is surrounded by a separate tendon sheath^2^ (*Vag. tendinis m. flexoris digit. medialis* ^1^, MDFTS) and fuses with the LDFT 1–3 cm distal to the tarsometatarsal joint.^3^ Communication between the MDFTS and tarsal sheath has previously been described.^2^

Traumatic injury to the medial aspect of the tarsus could result in tenosynovitis of the MDFTS, tearing of the MDFT, or penetration leading to subsequent septic tenosynovitis, for which endoscopy may be indicated. However, no reports exist on the endoscopic evaluation of the MDFTS. Needle endoscopy in horses has been described for various synovial structures^5^, ^6^, ^7^, ^8^, ^9^, ^10^, ^11^, ^12^ and could serve as a useful diagnostic tool for assessing the MDFT and MDFTS due to its small size.^13^ To the authors' knowledge, there are currently no descriptions of the endoscopic and computed tomographic (CT) anatomy of these structures. Additionally, the endoscopic approach to the MDFTS has not been documented.

The aims of the study were: (1) to describe the anatomy of the MDFTS by use of CT, endoscopic examination, and macroscopic examination by dissection; (2) to identify the portal locations for the endoscopic examination of the MDFTS; (3) to determine the visualization and limitations during endoscopic examination of the MDFTS with a 0°, 1.9 mm needle arthroscope and a conventional 30°, 4 mm arthroscope; and (4) to describe a clinical case of traumatic septic tenosynovitis of the MDFTS.

Based on preliminary data, we hypothesized that (1) the anatomy of the MDFTS would vary among individual horses, (2) a distal and proximal endoscopic portal would enable safe entry and a complete evaluation of the MDFTS and (3) evaluating the MDFTS would be more feasible with the needle arthroscope than with the conventional arthroscope.

## 2 MATERIALS AND METHODS

### 2.1 Study design and preparation

All cadaveric pelvic limbs (*n* = 12) were collected from horses that had been euthanized for reasons unrelated to the study and without any visible injuries to the hindlimbs. Limbs were disarticulated at the stifle in order to leave the origin of the medial digital flexor muscle at the medial tibial condyle as well as the lateral digital flexor muscle and the caudal tibial muscle at the lateral tibial condyle and the head of the fibula intact. Limbs were frozen at −7°C and thawed 48 h prior to use. Owner consent was obtained for the use of cadaveric limbs.

### 2.2 Anatomical study

Macroscopic dissection was performed to identify the anatomical structures that would affect portal placement (neurovascular bundles, mesotenons, flexor retinaculum) and compare their endoscopic appearance with the macroscopic appearance.

For the dissection of the limbs, the skin at the medioplantar aspect of the tarsus and metatarsus was removed with a no. 10 scalpel blade. The distal third of the tarsal sheath was opened, and the LDFT was transected and retracted distally. The point of fusion of the MDFT with the LDFT was recorded, and both sheath walls were checked for any fenestrations that would allow communication between the MDFTS and the tarsal sheath. Subsequently, the MDFTS was incised at the distomedial aspect and longitudinally opened in a proximal direction, thereby dissecting the plantar fascia and flexor retinaculum. The mesotenon anatomy and the presence and location of other intrasynovial structures were recorded.

### 2.3 Computed tomography

Limbs were clipped with a 40 mm blade, washed and positioned with the medial aspect facing upward. The first CT scan (16‐slice CT scanner; Brilliance CT Big‐Bore‐Oncology, Philips Medical Systems, Best, The Netherlands) was performed without contrast medium with 120 kV, 500 mAs, slice thickness of 0.8 mm and pitch of 0.438. The images were viewed using a soft tissue filter (window level [WL] 139 HU, window width [WW] 201 HU) and a bone filter (WL 1000 HU, WW 2400 HU).

The second CT examination included a contrast tenography of the MDFTS. Under ultrasonographic guidance (GE Logiq V2, GE Healthcare, Chalfont St. Giles, UK) using a linear probe (GE L6‐12‐RS probe), a 21‐gauge 40 mm needle was inserted into the tarsal sheath and MDFTS, just distal to the chestnut where the tarsal sheath and the MDFTS run directly next to each other, separated only by a very thin wall. This distal location was selected because significant fluid extravasation at the injection site was observed in the cadaveric limbs, with direct MDFTS distention at the medial aspect of the tarsus. Since the medial aspect of the tarsus was the area of interest for this CT examination, this injection site was deemed unsuitable as extravasation of contrast media in this area could compromise the assessment of the MDFTS. The extravasation would also complicate the subsequent endoscopic examination involving the placement of portals and the endoscopic procedure itself. Both structures were distended at this level through the same injection site as the communication between the MDFTS and the tarsal sheath has not been consistently reported. Attempting to inject the MDFTS solely in this distal region also resulted in distension of the tarsal sheath in the first limb, either by puncturing the thin separation with the needle or due to a preexisting communication between the structures. Consequently, we elected to distend both the MDFTS and tarsal sheath in all limbs to establish a consistent distention pattern, irrespective of the communication between the tarsal sheath and the MDFTS. The tarsal sheath and MDFTS were distended using Iohexol 300 mg/mL (Accupaque 300, GE Healthcare Buchler GmbH & Co. KG, Braunschweig, Germany) diluted 1:1 in a 0.9% saline solution (Fresenius Kabi AG, Bad Homburg, Germany) until maximally distended, determined by digital assessment of the distention just distal to the flexor retinaculum. Distention of the proximal aspect of the MDFTS was confirmed by ultrasound. Following the contrast injection, the limb was flexed and extended in 15 cycles to ensure even distribution of the contrast medium. The CT examination was repeated using the same protocol.

Images were evaluated retrospectively using a DICOM viewer (easyIMAGE, VetZ GmbH, Isernhagen, Germany) by two ECVS Diplomates (UD, NV) and one ECVS Resident (MPK). The following variables were recorded for the MDFTS: proximodistal length (cm), maximal cross‐sectional area (cm^2^) and location, minimal cross‐sectional area (cm^2^) and location, mesotenon presence, location and anatomical relationship, contrast filling defects, and the shape of the sheath.

### 2.4 Endoscopic examination of the MDFTS

All endoscopic examinations were conducted by the same ECVS Diplomate (NV). The location for potential portals for endoscopic examination was based on pilot CT examination and gross anatomical dissection. The approach had to be at a point with proper distention of the MDFTS and remain clear of the mesotenon and flexor retinaculum. An additional criterion was that both distal and proximal portals allow for evaluation of the entire length of the sheath.

Following CT, the same hindlimbs were used for endoscopy. To achieve this, the limb was placed on a surgery table with the limb in extension and the medial aspect facing upward.

In case of loss of distention between the CT and endoscopic examination, the tarsal sheath and MDFTS were distended again under ultrasonographic guidance in the same fashion as described for the contrast injection. Endoscopy of the MDFTS was initially performed using the needle scope (NanoScope, Arthrex Vet Systems, Munich, Germany). The first portal was placed at approximately one‐third of the distance between the medial malleolus and the chestnut at the point of maximum distention. A small stab incision was made with a no. 11 blade, and the needle scope trocar with a sharp obturator was advanced into the MDFTS under ultrasonographic guidance in a distad direction. Distention was maintained using a fluid pump (continuous wave 4 Arthroscopy Pump, Arthrex Vet Systems), with fluid pressure set at 100 mmHg initially and increased to 180 mmHg if necessary. After retroversion of the scope, a second portal was made proximal to the flexor retinaculum under endoscopic guidance to enable the evaluation of the proximal section of the sheath. An 18‐gauge needle was placed to allow fluid egress during examination.

As explained in the results section, two portals were necessary to examine the entire length of the MDFTS. The first portal allowed examination of the middle and distal segments of the MDFTS, while the second portal enabled examination of the proximal segment. Intrasynovial structures identified during dissection and CT examination were assessed for visibility during the endoscopic examination. Still images and videos were taken of all intrasynovial structures, with particular focus on the visibility of the communication between the tarsal sheath and the MDFTS.

A grading system was implemented based on Baldwin et al.^14^ to report visualization (0, no ability to visualize the intrasynovial structure; 1, incomplete/partial visualization of the intrasynovial structure; 2, complete visualization of the intrasynovial structure). Complete visualization was reserved for those structures, where >90% of the structure was visible endoscopically. This percentage was assessed subjectively and based on gross dissection. Extravasation was categorized as none, mild (not limiting endoscopic examination), moderate (some restriction to endoscopic examination), or marked (prohibiting completion of endoscopic examination). Obtained still images and videos were evaluated by two ECVS Diplomates (UD, NV) and an ECVS resident (MPK). Grading of the images was performed through consensus decision‐making.

Following the endoscopic examination using the needle arthroscope, the portal was enlarged to facilitate the insertion of the conventional arthroscope (30° 4.0 mm HD arthroscope, Arthrex Vet Systems). The endoscopic examination with the conventional arthroscope was conducted in the same manner as with the needle arthroscope.

After the endoscopic examination, all specimens were dissected to correlate the endoscopic findings with the macroscopic anatomy and to identify any iatrogenic damage.

### 2.5 Pilot study

Two hindlimbs were used for initial gross anatomical dissection only in order to get an understanding about the anatomy and anatomical landmarks of the MDFT and its MDFTS. Four additional hindlimbs underwent pilot CT and pilot endoscopy followed by gross anatomical dissection.

### 2.6 Main study

In six hindlimbs from two Warmbloods and one Quarter Horse, the CT, endoscopic examination and anatomical dissection were conducted in the same manner as established by the pilot study. The endoscopic examination was conducted solely with the needle scope since our pilot study revealed that using the conventional arthroscope for endoscopic examination was not feasible.

## 3 RESULTS

### 3.1 Anatomical study

**FIGURE 1:**
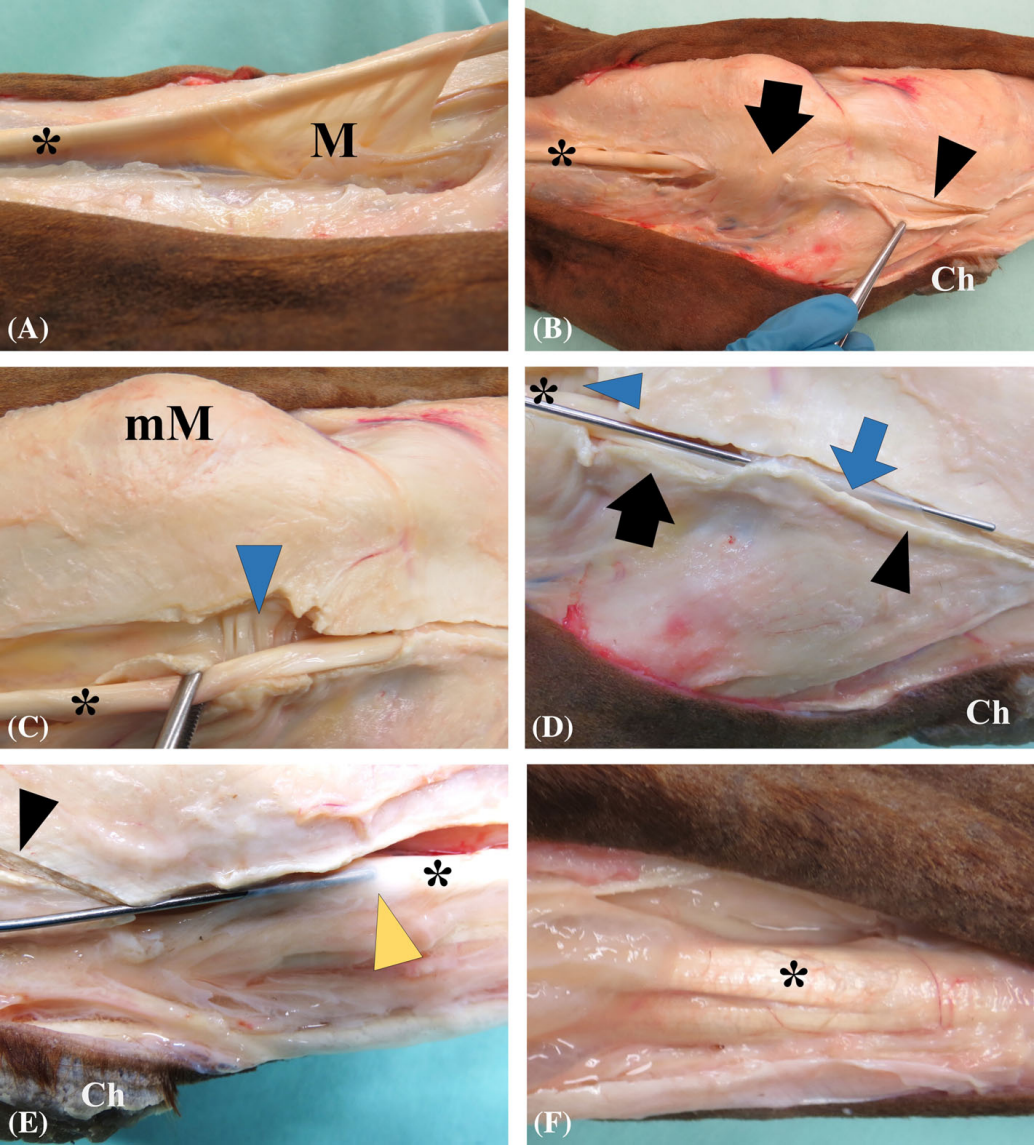
Anatomical dissection of the medial aspect of a left hindlimb, with proximal to the left and caudal/plantar to the bottom of each image. (A) The proximal sheath‐like mesotenon (M) is seen attached to the caudoaxial aspect of the medial digital flexor tendon (MDFT*). (B) The flexor retinaculum (black arrow) crosses over the MDFT where it courses through the malleolar groove. The plantar fascia is partially incised distally (black arrowhead). Ch = chestnut. (C) Transverse folds (blue arrowhead) of the axial wall of the medial digital flexor tendon sheath (MDFTS) at the distal aspect of the medial plantar pouch of the talocrural joint. mM = medial malleolus. (D) Metal probe inserted underneath the synovial plica (blue arrow) coursing around the MDFTS, distal to the transected flexor retinaculum (black arrow). (E) A metal probe inserted in the synovial plica consistently seen just distal to the chestnut with the tip of the probe placed in the blind sack (yellow arrowhead). (F) Fusion of the MDFT with the lateral digital flexor tendon (LDFT).

No pathology of the MDFT and MDFTS was noted on gross anatomical dissection of any limbs. The average proximodistal length of the MDFTS was 26.7 cm (range, 23–33 cm). In all limbs, a mesotenon was located at the proximal end of the MDFTS with either a caudoaxial (6/12) or cranioaxial (6/12) orientation, and a mean length of 5.3 cm (range, 2–8 cm) (Figure 1A). Moving distad, a transversely oriented ligament, the flexor retinaculum (*Retinaculum flexorum* ^1^), overlaid the MDFTS as it passed through the malleolar groove (*Sulcus malleolaris* ^1^) of the medial malleolus (Figure 1B). The axial wall of the MDFTS exhibited small transverse folds as it ran directly over the distal aspect of the plantaromedial outpouching of the talocrural joint (Figure 1C). Distal to these transverse folds, a synovial plica attached to the MDFTS wall on the caudal aspect surrounded the MDFT on the dorsal aspect without direct attachment to the tendon (Figure 1D). At the level of the chestnut, there was another prominent synovial plica surrounding the MDFT, with caudal attachments forming a dorsal cul‐de‐sac within the MDFTS (Figure 1E). Anatomical variations of these synovial plicae were observed, with differences in thickness, proximodistal length, and the presence of fenestrations within the plicae. At the distal end of the MDFTS, thin translucent tissue separated the MDFTS from the tarsal sheath. Larger fenestrations within this tissue were macroscopically visible in 2/12 hindlimbs originating from the same horse, creating a visible communication between the MDFTS and the tarsal sheath in these limbs. Fusion of the MDFT with the LDFT occurred either on the dorsomedial or medial aspect, 5–7 cm distal to the TMT joint (Figure 1F).

### 3.2 Computed tomography

**FIGURE 2:**
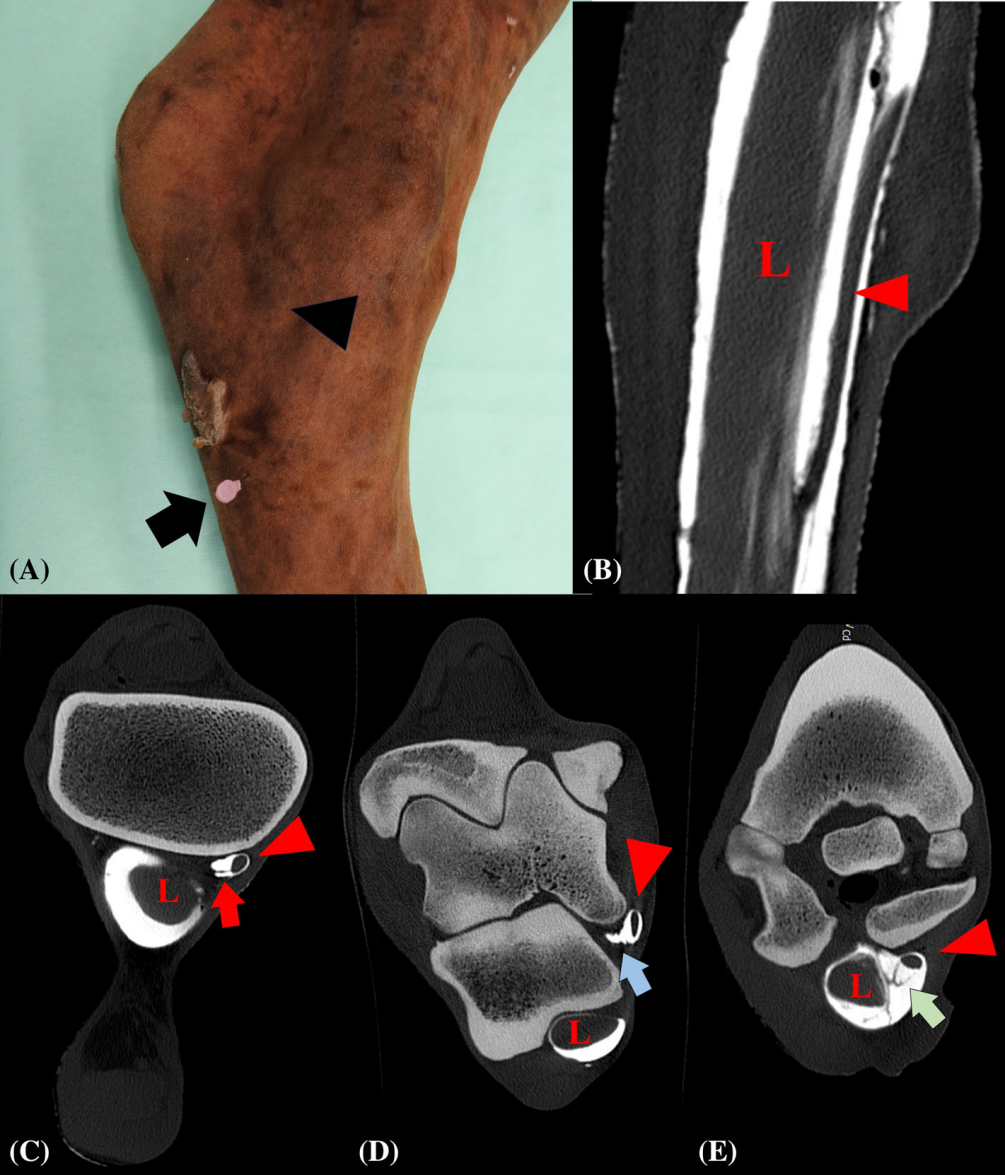
(A) Medial aspect of a left hindlimb depicting the location for contrast injection into the tarsal sheath (TS) applied in the current study (black arrow) and the point of maximal palpable distention of the medial digital flexor tendon sheath (MDFTS, black arrowhead). (B) Frontal computed tomographic (CT) image showing the lateral digital flexor tendon (L) within the TS and the medial flexor tendon (MDFT) within the MDFTS (red arrowhead). (C) Transverse CT image of the proximal MDFTS showing the proximal mesotenon of the MDFT (red arrow). (D) Transverse CT image of the middle section of the MDFTS showing the outpouching of axial wall of MDFTS (blue arrow) into the distal aspect of the medial plantar pouch of the talocrural joint. (E) Transverse CT image of the distal MDFTS showing a filling defect within the MDFTS surrounding the MDFT (green arrow), corresponding with the synovial plica that forms an intrasynovial cul‐de‐sac just distal to the chestnut.

No pathology of the MDFT was noted on the CT examination in any of the limbs. Ultrasound‐guided distention was successful on the first attempt (9/10) or second attempt (1/10). A volume of 80–120 mL of contrast medium was used to distend the MDFTS and tarsal sheath (Figure 2A). In all limbs, the contrast medium within the MDFTS was visible throughout the entire sheath. Distention of the MDFTS was good in all limbs except one. This was confirmed by the amount of contrast visible in the sheath on the CT images.

Regarding the CT findings (Data S1), the mesotenon at the proximal aspect of the MDFTS was identified in all CT examinations in caudoaxial (5/10) or cranioaxial (5/10) orientation (Figure 2B,C). The maximal cross‐sectional area (mean 0.75 cm^2^, range, 0.5–1.5 cm^2^) was located immediately distal to the flexor retinaculum in all limbs. This area coincided with the palpable site of maximal distention, and was therefore chosen as the location for the first endoscopic portal. Furthermore, there was an axial outpouching of the MDFTS at the level of the distal aspect of the plantaromedial outpouching of the TCJ, corresponding with the transverse folds observed during dissection (Figure 2D). The minimal cross‐sectional area (mean 0.37 cm^2^, range, 0.2–0.6 cm^2^) of the MDFTS was consistently found where the MDFT courses through the groove of the proximal tubercle of the talus, beneath the flexor retinaculum. The distal synovial plica with a cul‐de‐sac at the level of the chestnut was identified as a filling defect in all horses (Figure 2E). Fusion of the MDFT with the LDFT was located either on the dorsomedial or medial aspect, 5–7 cm distal to the TMT joint (Figure 2F).

### 3.3 Pilot endoscopic examination of the MDFTS

The first portal was created at the point of maximal palpable distention, as previously described. This corresponds to the anatomical landmarks of approximately one third of the distance between the medial malleolus and the chestnut. This initial portal allowed for the examination of the middle and distal aspects of the MDFTS. The proximal section of the sheath could not be assessed through this portal due to the limited length of the scope. However, even with a longer scope, the curvature of the sheath over the medial aspect of the tarsus, combined with the restrictions of the flexor retinaculum would impair examination of the proximal segment. For this reason, a second portal was made proximal to the flexor retinaculum under endoscopic guidance to evaluate the proximal segment of the sheath.

Following examination with the needle scope, the portal was enlarged to allow access to the MDFTS with the conventional arthroscope. Endoscopic entry was successful; however, maneuvering the scope within the sheath proved impossible due to the small size of the sheath. Even with increased force, movement was severely restricted and resulted in significant iatrogenic damage to the tendon and MDFTS wall. Furthermore, it was not possible to advance the scope in a proximal direction because of constriction by the flexor retinaculum. Dissection following endoscopy confirmed iatrogenic damage to the MDFT. Therefore, further attempts with the conventional scope were ceased. This technique was not deemed to be a feasible option for endoscopic examination in clinical patients, leading to its exclusion from the main study.

### 3.4 Needle endoscopic examination of the MDFTS

Ultrasound‐guided endoscopic entry was successful on the first attempt in all cases. It was possible to maneuver the needle scope around the MDFT within its sheath during the examination for all limbs. Moderate extravasation developed during the examination in all limbs.

In all segments, the MDFT could be completely visualized. The same applied to the MDFTS wall, except for the MDFTS wall in the proximal segment obscured by the mesotenon.

**FIGURE 3:**
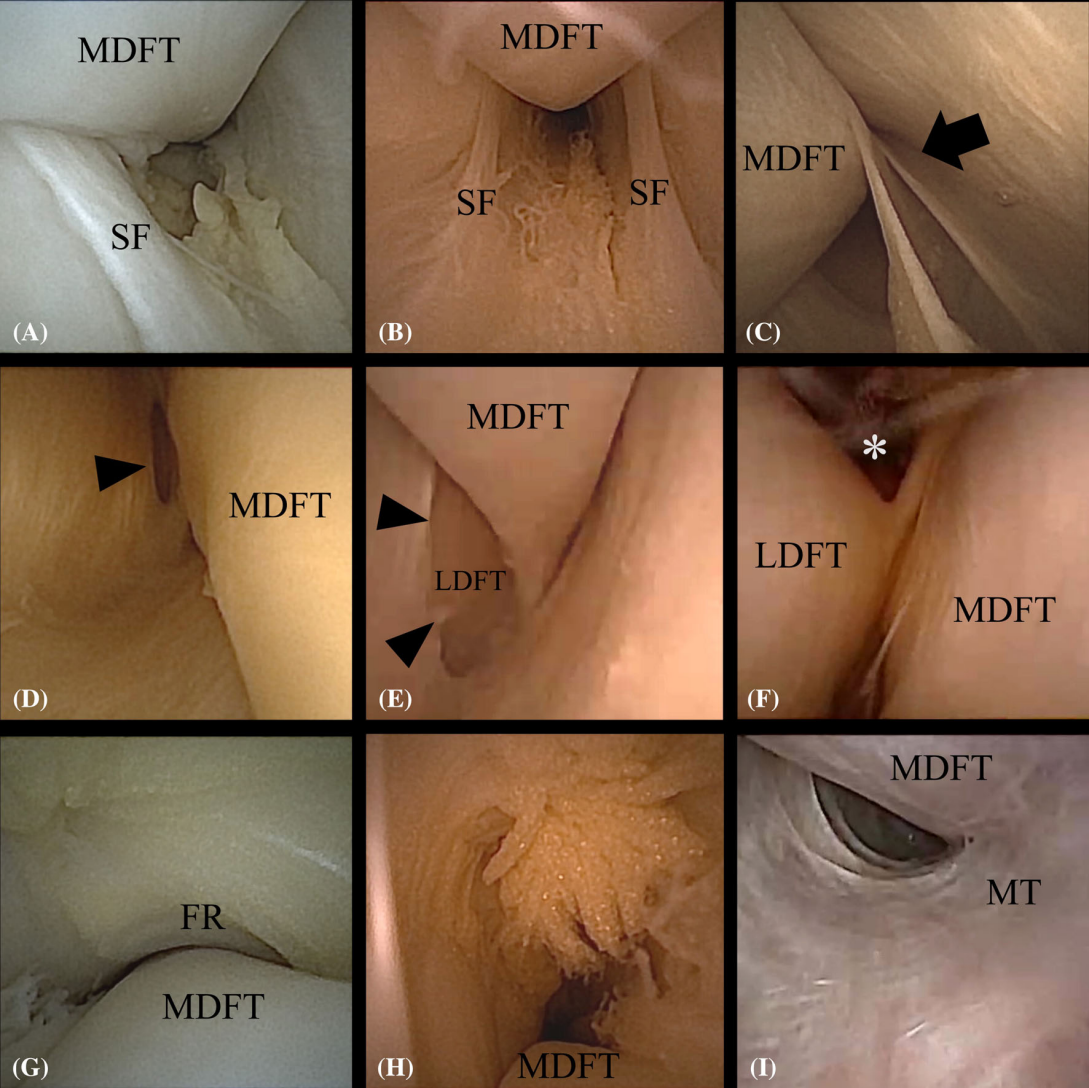
Endoscopic images of the medial digital flexor tendon sheath (MDFTS) obtained with a needle scope. (A) Synovial plica (SF) surrounding the medial digital flexor tendon (MDFT) distal to the flexor retinaculum. (B, C) Distal synovial plica (SF) forming a dorsal intrasynovial cul‐de‐sac (black arrow) distal to the chestnut. (D, E) Distal MDFTS showing the communication between the MDFTS and the tarsal sheath as fenestration in the synovial membrane (arrowheads). (F) Slightly more distally, the fusion of the MDFT and the lateral digital flexor tendon (LDFT) can be seen, as well as the continuation of the MDFTS into the tarsal sheath (*). (G) Endoscopic view looking proximal at the constriction of the MDFTS by the flexor retinaculum (FR) where it courses over the MDFTS in the malleolar groove. (H) Endoscopic visualization of the villi proliferation in the middle section of the MDFTS proximal to the FR and at the level of the transverse folds in the axial wall of the MDFTS. (I) Endoscopic visualization of the proximal MDFTS with the mesotenon (MT) attached to the axial wall.

Beginning with the distal segment of the sheath, going distad from the first portal, the proximal synovial plica was immediately visualized distal to the first portal (Table 1, Figure 3A). Visualization of this structure was incomplete in 9/10 limbs due to its proximity to the portal. In one limb, this structure could not be seen because the portal was created through this plica. The more distal synovial plica, forming a dorsal cul‐de‐sac, was fully visualized in all limbs (Figure 3B,C). Two limbs exhibited fenestrations in this plica on both the axial and abaxial sides. Following the caudal border of the MDFT distally, the membrane that divided the MDFTS from the tarsal sheath was visible in all horses. In two limbs, an oval fenestration was identified in this structure on the caudal aspect of the MDFTS (Figure 3D,E), confirming communication between the tarsal sheath and the MDFTS. In one of these limbs, it was possible to enter the tarsal sheath through this fenestration allowing for visualization of the fusion between the MDFT and LDFT (Figure 3F). Evaluating the most distal aspect of the MDFTS necessitated the full length of the needle scope.

**TABLE 1:** Endoscopic visibility score^a^ of the intrasynovial structures within the medial digital flexor tendon sheath with a 0° needle arthroscope.

Horse	Right or left	Number of attempts to enter with needle scope	Proximal aspect		Middle aspect		Distal aspect			MDFT	Extravasation
			Proximal end of MDFTS	Mesotenon	Transverse folds in MDFTS wall	Synovial plica	Synovial plica with dorsal cul‐de‐sac	Fenestration to tarsal sheath	Distal end of MDFTS		
Pilot 1	L	1	0	1	2	1	2	0	0	2	2
Pilot 2	R	1	0	1	2	1	2	0	0	2	2
Pilot 3	R	1	1	1	2	1	2	0	0	2	2
Pilot 4	L	1	1	1	2	2	2	2	2	2	2
Main study 1	R	1	1	1	2	1	2	0	0	2	2
Main study 2	L	1	1	1	2	2	2	0	2	2	2
Main study 3	R	1	0	1	2	0	2	2	2	2	2
Main study 4	L	1	1	1	2	0	2	2	2	2	2
Main study 5	R	1	1	1	2	1	2	0	2	2	2
Main study 6	L	1	1	1	2	2	2	0	0	2	2

Abbreviations: MDFT, medial digital flexor tendon; MDFTS, medial digital flexor tendon sheath.

^a^ Visualization scores of the intrasynovial structures: 0, no visualization; 1, incomplete/partial visualization; 2, complete visualization. Extravasation was scored as following: 0, none; 1, mild; 2, moderate; 3, marked.

After examining the distal segment of the MDFTS, the scope was retracted and redirected in a proximad direction to inspect the mid‐section. Passing beneath the flexor retinaculum (Figure 3G), the transverse folds in the MDFTS wall overlying the TCJ were visible in all limbs (Figure 3H). As previously stated, the MDFT could be fully visualized in this section.

After creating the second portal to enable examination of the proximal segment, aspects of the proximal mesotenon were visible in all cases (Figure 3I). However, complete visualization and assessment of the direction of the mesotenon were not possible in any of the limbs. The proximal end was visible in all limbs except one. No iatrogenic damage was observed in the dissections after needle endoscopy.

### 3.5 Case presentation

**FIGURE 4:**
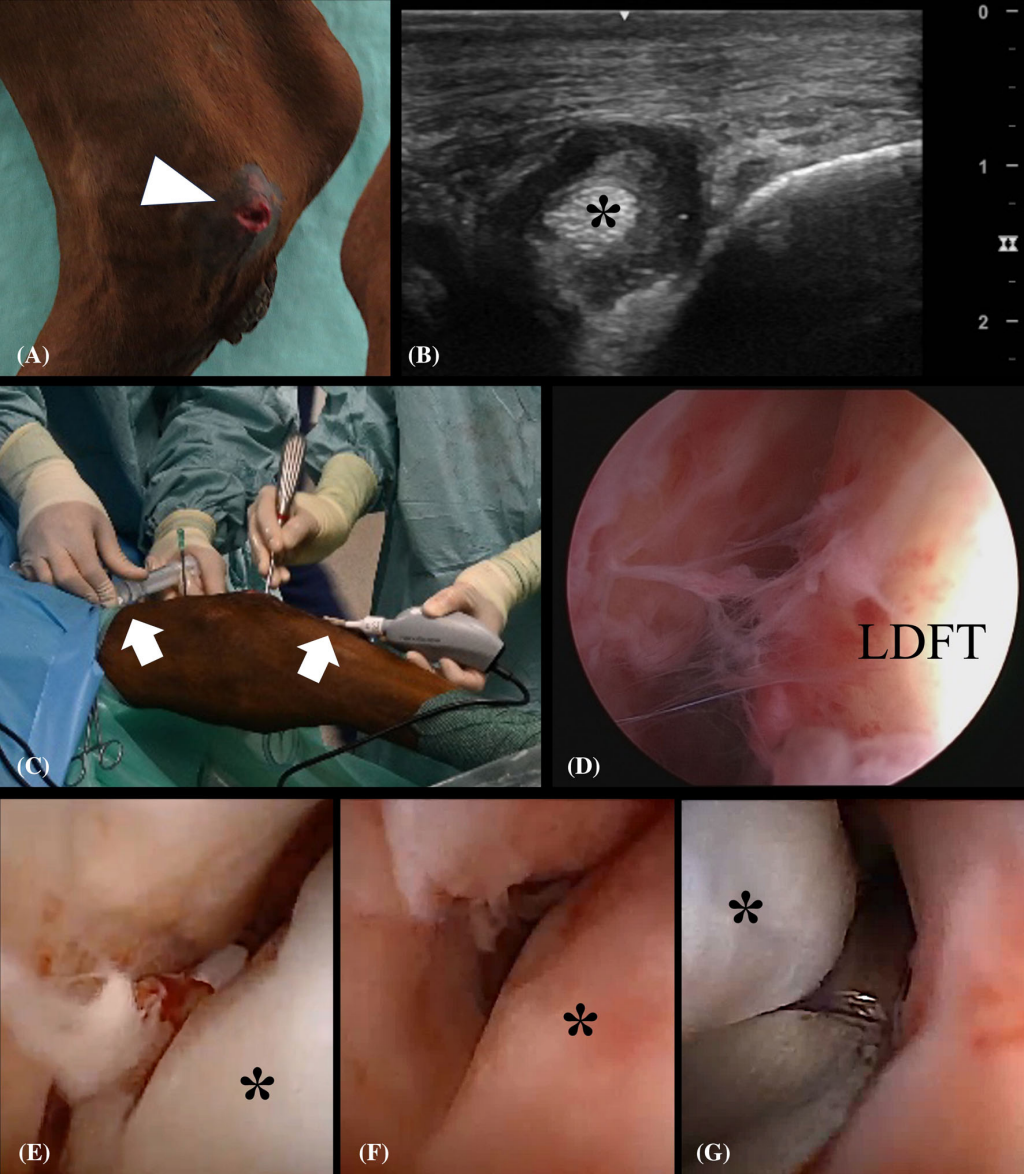
Images illustrating a clinical case of a septic medial digital flexor tendon sheath (MDFTS) of the right hindlimb (A) due to a traumatic laceration (white arrowhead) at the medial aspect of the tarsus. (B) Transverse ultrasonographic image of the medial aspect of the tarsus showing the medial digital flexor tendon (*) within a distended MDFTS with increased heteroechogenic content. (C) Intraoperative clinical image showing the distal and proximal portals (white arrows) for endoscopic lavage of the MDFTS using a needle scope. (D) Endoscopic image of the tarsal sheath obtained with a conventional 4 mm arthroscope showing hyperemic synovium and moderate fibrinous deposition. (E, F) Images of the MDFT (*) within the MDFTS obtained by needle endoscopy showing moderate fibrinous deposition with thickening and hyperemia of the synovium. (G) Debridement of the MDFTS was performed with a small curette through the laceration.

An 8‐year‐old Irish Sport Horse gelding (570 kg) was referred to the Clinic for Horses because of a 4/5 (AAEP scale) right hindlimb lameness approximately 24 h after the horse stumbled and fell on the street. The horse presented with a transverse skin laceration of approximately 3 cm just caudal to the medial malleolus (Figure 4A). There was a moderate to marked distention of TCJ and the tarsal sheath. Synovial fluid analysis of TCJ and tarsal sheath showed a white blood cell (WBC) count of 200 cells/μL and 46,000 cells/μL, and a total protein (TP) of 30 and 52 g/L, respectively. The MDFTS was not specifically investigated at this stage. The horse received 6.6 mg/kg gentamicin sulfate IV, 10 mg/kg amoxicillin sodium IV and 1.1 mg/kg flunixin meglumine IV preoperatively. Under general anesthesia, the tarsal sheath was lavaged with 12 L of Ringer's solution using a routine endoscopic technique and the wound was debrided. Although the wound did not directly overlie the tarsal sheath, there was continuous fluid egress through the wound during the lavage. Therefore, involvement of the MDFTS was suspected, with fluid egress facilitated by the communication between the tarsal sheath and the MDFTS. The fusion of the MDFT with the LDFT was seen at the level of the chestnut, yet the communication between the tarsal sheath and MDFTS could not be visualized. The distal tarsal sheath was very hemorrhagic with fibrinous deposition. The MDFTS was not endoscopically examined separately during this surgery because the surgeon felt this structure was lavaged sufficiently by flushing the tarsal sheath. The portals were closed in a routine fashion and the wound was closed in a single interrupted vertical mattress pattern (Nylon 1 USP). A total of 2 g of amikacin were administered in the tarsal sheath and a two‐layer full‐limb bandage was applied. Microbiologic analysis of samples from the wound and the synovia of the tarsal sheath taken on admission revealed a monoculture of *Streptococcus equi zooepidemicus*.

Postoperatively, the horse showed a persistent lameness and synoviocentesis of the tarsal sheath revealed a TP of 72 g/L and WBC count of 23, 900 cells/μL 2 days after surgery. On ultrasound, the MDFTS was distended with heteroechogenic fluid and suspected fibrin deposition (Figure 4B), but a synovial sample was not obtained. A second surgery was performed under general anesthesia in right lateral recumbency with the limb in extension. Sutures from the first surgery were removed and wound debridement was repeated. Under ultrasonographic guidance, a needle scope trocar was inserted in both the proximal and distal recess of the MDFTS (Figure 4C). Moderate fibrinous deposition with thickening and hyperemia of the synovium was seen. There was egress of lavage fluid through the wound, and the MDFTS was debrided through the wound (Figure 4E–G). The tarsal sheath was lavaged by making a first portal at the proximal aspect of the *Sustentaculum tali* and reusing the portals from the first surgery. The synovium from the tarsal sheath was hyperemic with moderate fibrinous deposition (Figure 4D). Under endoscopic visualization, a 20G catheter (Perifix Epidural Anesthesia Catheter, B. Braun Medical BV, Melsungen, Germany) was placed intrasynovially in the proximal recess of the MDFTS and one was placed in the proximal aspect of the tarsal sheath. Portals were routinely closed and the wound was closed with near‐far‐far‐near and vertical matrass sutures (Nylon 1 USP). Intrasynovial administration of 500 mg amikacin was performed through both intrasynovial catheters immediately postoperatively.

This was repeated for five consecutive days once daily, followed by removal of the catheters. Intravenous antibiotics were continued for 7 days postoperatively and non‐steroidal anti‐inflammatories for 5 days. The two‐layer full‐limb bandage was changed every 2–3 days. Sutures were removed 14 days postoperatively, and the wound healed per primam. Initially, the horse did show shortening of the protraction phase of the right hindlimb at the walk until 18 days after the second surgery. The horse was discharged 21 days postoperatively. Follow‐up by telephone conversation with the owner 20 months after surgery revealed that the horse has been sound since discharge from the hospital, was being ridden five to six times a week and being primarily used as hunting horse.

## 4 DISCUSSION

The present study investigated the gross and computed tomographic anatomy, in addition to the endoscopic examination of the MDFTS. One of our main findings was that several intrasynovial structures, such as the proximal mesotenon and the synovial plicae, were consistently present within the MDFTS, although their appearance varied between limbs. On the other hand, communication between the MDFTS and tarsal sheath varied among individual horses. Consequently, the data supported our first hypothesis that the anatomy of the MDFTS would vary among horses. A proximal and distal portal were established for the endoscopic examination of the MDFTS using a needle scope. Although endoscopic entry with a conventional arthroscope was achieved, examination with this endoscope was not possible due to poor maneuverability within the sheath. Therefore, the data supported our second and third hypotheses that the portals would enable safe entry and evaluation of the MDFTS and that evaluation of the MDFTS is more feasible with the needle arthroscope.

The selected first portal location was situated at the level of the maximum cross‐sectional area of the MDFTS, approximately one‐third of the distance between the medial malleolus and chestnut. This portal provided good visualization of the distal aspect of the MDFTS. A potential drawback of this portal location is its proximity to the synovial plica. In one limb, the portal was created through this synovial plica, which could have caused hemorrhage in the live horse. Nonetheless, this portal location at the point of maximum distension remains suitable as the initial portal to enable the entry into this small synovial structure. If the most distal aspect of the MDFTS is the main point of interest, a longer scope may be advantageous. To facilitate examination of the proximal aspect, a second portal was placed proximal to the flexor retinaculum under needle scope guidance. We found that a complete examination of the MDFTS using only the first portal would be challenging due to the length of the scope, poor maneuverability beneath the flexor retinaculum, and the curved trajectory of the sheath over the tarsus. The placement of additional portals was not evaluated in this study.

An endoscopic examination without iatrogenic damage to the MDFT was only possible with the needle scope. The diameter of a conventional arthroscope sleeve that is often used for equine endoscopy is 5.9 mm, compared to 2.4 mm for the NanoScope. Based on the size of these scopes, it should be concluded that the size conventional arthroscope is too large to enable examination at the smallest section of the MDFTS, at the groove of the proximal tubercle of the talus. A limitation of the needle scope was the 0° direction of view. Small bore conventional endoscopes used in small animal surgery or needle scopes with a working angle of 10° and 30° that are now available for veterinary use might further improve the evaluation of the MDFTS.

Evaluating the anatomy of the MDFTS, two synovial plicae were consistently recognized in all limbs. It has been suggested that the reduplication of the synovium may allow a tendon to move within its tendon sheath.^15^ This could explain the presence of these plicae in the MDFTS at the level of the chestnut, where its trajectory changes from a proximodorsal‐distoplantar to a proximodistal direction at the level of the tarsometatarsal joint. The clinical relevance of these synovial plicae is questionable and cannot be clarified by the current results. Another anatomical feature was the communication between the MDFTS and tarsal sheath which varies among horses.^16^ In the current study, communication between the MDFTS and the tarsal sheath was visualized in only 2/12 limbs from the same horse, while it was not found in the other hindlimbs. There may have been small fenestrations in the thin distal membrane between the two sheaths that were not observed during dissection and endoscopy. During distention of the MDFTS and the tarsal sheath, the needle may have entered the MDFTS through the thin synovial membrane at the injection site. This ambiguity may be regarded as a significant limitation of the current study. However, assessing the prevalence of communication between the tarsal sheath and MDFTS was not the objective and many more specimens would have been necessary to reliably determine the prevalence and variation in this communication. Due to the extravasation in these cadaveric limbs on the medial aspect of the tarsus when directly distending the MDFTS, we chose this distal location for distention. For clinical cases where the MDFTS is the region of interest, we recommend performing synoviocentesis of the MDFTS alone. For this, the first portal site just distal to the flexor retinaculum would be most suitable.

The communication between the MDFTS and the tarsal sheath is clinically significant when treating septic tenosynovitis of either the tarsal sheath^17^ or the MDFTS. This is illustrated by the clinical case in which the tarsal sheath developed septic tenosynovitis due to a wound at the level of the MDFTS. Septic tenosynovitis of the MDFTS is an uncommon diagnosis but has been reported as a result of a wound on the medial aspect of the tarsus.^18^ In the present study, lavage of the tarsal sheath alone did not resolve the septic tenosynovitis. Especially within tendon sheaths, lavage fluids frequently flow over or past intrasynovial structures, leaving behind pockets of debris.^19^ The presence of numerous intrasynovial structures within the MDFTS, combined with our clinical case, suggests that endoscopically guided lavage and debridement is worthwhile for the treatment septic tenosynovitis of the MDFTS. The proximity of the MDFTS to the distal aspect of the plantaromedial outpouching of the TCJ may also be of significance in case of trauma in this anatomic region. Another indication for examining the MDFTS is a fracture of the proximal tubercle of the talus.^20^, ^21^, ^22^ However, a surgical intervention for this indication could be challenging due to the small size of the synovial structure.

Alongside the previously mentioned limitations, the ex ‐vivo study design should be regarded as the main limitation. As a result, it could not be assessed whether damage to intrasynovial structures might have caused intrathecal hemorrhage and associated poor visualization. Additionally, this may have contributed to the extravasation that occurred during the examination, potentially leading to reduced visibility in the proximal recess of the MDFTS in this study, after starting the examination going distad. It is also possible that using the chestnut as a reference in the cadaveric limb may differ from the position of the chestnut in an intact limb, even though the skin at the level of the tarsus was judged to be in a physiological position. Other limitations included the small number of cadaveric hindlimbs, the use of clinically normal limbs and the use of different breeds, precluding definitive conclusions regarding the distribution of anatomical variations of the MDFTS.

In conclusion, needle arthroscopy facilitates the endoscopic examination of the MDFTS and a complete endoscopic examination requires at least two portals. This may be indicated for the treatment of septic tenosynovitis of the MDFTS and/or the tarsal sheath. The current study revealed multiple intrasynovial structures that should be considered during portal placement and lavage of the sheath. The communication between the MDFTS and the tarsal sheath could not be consistently visualized and there was considerable individual variation among the horses. Therefore, studies including more specimens are necessary to further elucidate the anatomy of this region.

## AUTHOR CONTRIBUTIONS

Kerbert MP, DVM: Contribution to study design, study execution, data analysis and interpretation, preparation and final approval of the manuscript. Delling U, MS, DACVS (Large Animal), DECVS: Contribution to study design, study execution, data analysis and interpretation and final approval of the manuscript. Verhaar N, DVM, PhD, DECVS: Contribution to study design, study execution, data analysis and interpretation, preparation and final approval of the manuscript.

## CONFLICT OF INTEREST STATEMENT

No conflict of interest to declare.

## Supporting information


**Data S1.** Supporting Information.

## Data Availability

The data supporting the findings of the study are available upon request.
