# Supplementary material for: Gross anatomy, computed tomographic contrast tenography, and needle endoscopy of the equine medial digital flexor tendon sheath
Source: Vet Surg. 2025 Apr 14;54(6):1133–44. doi: 10.1111/vsu.14263 (PMC12344221; doi:10.1111/vsu.14263)
Supplement: Supplementary file 1 — Data S1. Supporting Information. [file VSU-54-1133-s001.docx]

**Supplementary information**

**S1**: Measurements and anatomical findings of the MDFTS on CT examination.

| Horse | Right or left | Proximodistal length (cm) | Max. CSA (cm^2^) | Min. CSA (cm^2^) | Mesotenon orientation | Location of insertion of the MDFT on LDFT (in cm distal to TMT) |
| --- | --- | --- | --- | --- | --- | --- |
| Pilot 1 | L | 26 | 0.6 | 0.2 | caudoaxial | dorsomedial (5) |
| Pilot 2 | R | 26 | 0.5 | 0.3 | cranioaxial | medial (5) |
| Pilot 3 | R | 29 | 0.8 | 0.5 | cranioaxial | medial (6) |
| Pilot 4 | L | 28 | 0.8 | 0.4 | caudoaxial | dorsomedial (4) |
| Main study 1 | R | 30 | 1.5 | 0.6 | caudoaxial | medial (5) |
| Main study 2 | L | 23 | 0.5 | 0.2 | cranioaxial | medial (6) |
| Main study 3 | R | 23 | 0.5 | 0.3 | cranioaxial | medial (6) |
| Main study 4 | L | 24 | 0.6 | 0.3 | cranioaxial | dorsomedial (4.5) |
| Main study 5 | R | 25 | 0.8 | 0.4 | caudoaxial | dorsomedial (5) |
| Main study 6 | L | 33 | 0.9 | 0.5 | caudoaxial | dorsomedial (4) |
| Mean |  | 26.7 | 0.75 | 0.37 |  |  |

MDFTS = medial digital flexor tendon, max. CSA = maximal cross-sectional area, min. CSA = minimal cross-sectional area,

MDFT = medial digital flexor tendon, LDFT = lateral digital flexor tendon.
